# Pre and post introduction of pneumococcal conjugate vaccines in children in Eastern Mediterranean Region and effect on antimicrobial resistance, a narrative review

**DOI:** 10.3389/fmicb.2026.1735082

**Published:** 2026-02-06

**Authors:** Basma M. Saleh, Osama Mere, Seham Elmrayed

**Affiliations:** 1 Institute of Human Health and Human Ecology, The American University in Cairo, Cairo, Egypt; 2 World Health Organization, Eastern Mediterranean Region Office, Cairo, Egypt

**Keywords:** antimicrobial resistance, children, Eastern Mediterranean Region, multi-drug resistance, pneumococcal vaccine, surveillance

## Abstract

**Background:**

Antimicrobial resistance (AMR) is a pressing public health challenge in the Eastern Mediterranean Region (EMR), with *Streptococcus pneumoniae* being a major contributor due to its role in invasive pneumococcal disease (IPD). Pneumococcal conjugate vaccines (PCVs) have shown promise in reducing vaccine-type IPD and AMR, yet significant barriers persist.

**Objective:**

This review examines the impact of PCVs on AMR, serotype distribution, and vaccine coverage in pediatric populations within the EMR, emphasizing the interplay between vaccination strategies and AMR trends.

**Content:**

The introduction of PCVs, particularly PCV13, has led to reductions in vaccine-type IPD and AMR in several EMR countries. For example, post-PCV13 penicillin susceptibility in Saudi Arabia improved from 37 to 100% in invasive isolates, and Lebanon reported significant reductions in vaccine-type AMR. However, challenges such as inconsistent vaccination coverage (e.g., 76% in Yemen, absent in Egypt), socio-political instability, and the emergence of non-vaccine serotypes (NVTs) as per shown in the 73.5% of isolates in Omani children and the high multidrug resistance (MDR) rates (e.g., 85.7% in Yemen) undermine these benefits. Serotype replacement phenomena, driven by ecological shifts, are increasingly contributing to disease burden and AMR. While PCVs reduce the need for antibiotics and limit selective pressures driving resistance, high rates of MDR and inadequate antibiotic stewardship remain concerns. Strengthened vaccination programs, high-valency vaccines, and robust surveillance systems are critical to addressing these challenges.

**Conclusion:**

PCVs play a pivotal role in mitigating AMR and reducing pneumococcal disease burden in the EMR. However, suboptimal vaccine coverage, NVT emergence, and persistent AMR highlight the need for comprehensive strategies, including enhanced coverage of higher valency vaccines, robust surveillance, targeted public health interventions, strengthened antibiotic stewardship, and improved regional collaboration. Addressing these issues can significantly reduce the AMR burden and improve health outcomes across the region.

## Introduction

*Streptococcus pneumoniae* is a pathogen that cause invasive pneumococcal diseases (IPD) and community acquired pneumonia resulting in pneumonia, septicemia, and meningitis. IPD is a leading contributor to global morbidity and mortality, especially in children under 5 years of age. According to the World Health Organization (WHO), Children under 5 account for 30.56% of all AMR attributable deaths globally as per 2021 GRAM (Global Burden of Bacterial Antimicrobial Resistance) data. In the EMR specifically, pneumococcal diseases remain a major cause of morbidity and mortality. A 2018 estimate indicate that, over one million children in developing countries die annually from pneumococcal diseases, despite the availability of vaccines for prevention (Azarsa et al., 2019; Cillóniz et al., 2018; Global Research on Antimicrobial Resistance [GRAM], 2024).

The primary treatment for pneumococcal infections is antibiotic including β-lactams and macrolides as first options and fluoroquinolones as secondary options. specific choices guided by syndrome (e.g., meningitis vs. pneumonia), local resistance patterns, and patient factors. β-lactams (e.g., penicillin, amoxicillin, ceftriaxone) and macrolides are common first-line options for non-meningeal infections, while meningitis often requires higher-dose or combination therapy including third-generation cephalosporins and vancomycin due to resistance concerns. Fluoroquinolones may be used in adults but are generally not first-line in children. The overuse of antibiotics has driven the emergence of resistant strains, such as penicillin-non-susceptible *S. pneumoniae* (PNSP), causing a global threat (Azarsa et al., 2019; Cillóniz et al., 2018; Global Research on Antimicrobial Resistance [GRAM], 2024).

Preventing *Streptococcus pneumoniae* infections and their spread in young children is an important goal of vaccination. In 2007, the WHO recommended incorporating the 7-valent pneumococcal conjugate vaccine (PCV7) into national immunization programs. PCV7 targets serotypes 4, 6B, 9V, 14, 18C, 19F, and 23F. Later, higher-valence vaccines were introduced: PCV10, which adds serotypes 1, 5, and 7F, and PCV13, which further adds serotypes 3, 6A, and 19A. PCVs not only protect vaccinated individuals but also reduce bacterial transmission, creating herd immunity and lowering the overall incidence of IPD (Nasereddin et al., 2013).

The replacement of vaccine-targeted serotypes with non-vaccine serotypes has been observed in both nasopharyngeal carriage and invasive pneumococcal disease following PCV implementation. Additionally, increases in non-susceptibility or resistance have emerged among non-vaccine serotypes in certain contexts. The incomplete understanding of the overall impact of these shifts on circulating pneumococcal populations has hindered comprehensive evaluations of the role of PCVs in mitigating antimicrobial resistance (AMR) (Andrejko et al., 2021).

Serotype replacement following PCV introduction is well-documented globally. For example, Analysis of IPD data from 10 European countries over 8 years of PCV programs with PCV10 and PCV13 revealed significant trends. Between the PCV7 period and 2018, IPD incidence decreased by 42% in children < 5 years, 32% in individuals aged 5–64 years, and 7% in those ≥ 65 years. However, non-PCV13 serotype IPD incidence increased substantially across all age groups, ranging from 63 to 111%, with the largest proportional increase observed in children. In regions with high PCV10/PCV13 uptake, non-PCV13 incidence in children rose by 223%, compared to 56% in areas with moderate uptake. While surveillance capacity and healthcare contexts differ between Europe and the EMR, these data underscore the importance of monitoring for similar replacement phenomena and their potential impact on AMR in the EMR following PCV introduction (Hanquet et al., 2022).

To provide an overview of pneumococcal conjugate vaccine (PCV) introduction across the Eastern Mediterranean Region, Table 1 summarizes different vaccine introduction timelines within the catch-up project in WHO-EMRO, types used, and policy shifts up to the fourth quarter of 2025, based on comprehensive regional immunization data.

**TABLE 1:** Vaccine introduction status within Eastern Mediterranean Region.

Country	Hep B BD	PCV	ROTA	DPT booster	Rubella	Influenza	HPV	Meningitis	Hep A	Varicella	Malaria	TCV	Yellow fever
Afghanistan	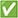	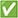	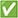	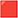	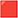	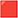	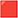	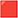					
Bahrain	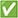	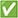	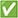	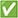	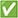	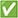	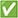	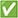	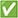	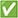			
Djibouti	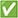	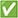	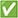	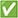	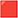	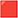	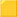 Q4 2025	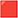					
Egypt	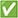	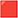	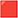	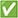	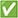	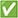	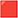	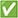					
Iran	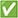	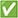	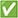	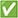	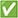	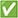	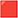	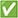		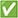			
Iraq	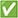	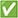	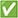	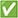	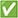	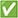	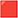	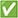					
Jordan	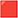	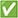	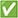	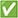	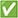	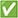	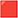	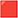	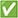				
Kuwait	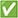	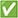	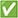	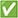	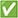	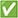	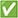	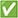	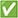	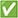			
Lebanon	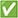	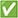	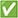	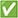	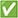	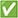	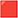	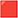					
Libya	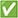	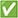	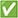	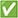	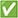	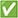	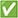	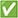	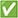	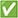			
Morocco	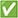	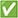	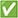	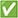	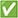	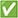	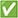	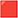					
Oman	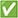	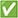	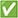	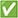	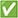	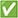	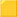 Q4 2025	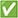		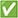			
Pakistan	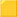 Partial	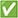	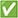	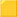 Partial	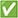	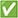	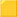 Q3 2025	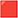				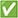	
Palestine	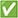	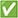	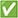	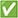	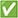	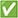	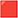						
Qatar	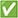	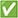	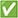	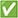	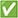	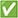	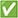	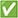	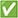	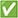			
Saudi Arabia	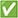	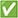	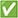	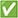	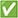	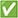	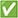	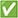	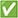	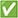			
Somalia	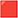	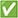	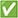	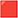	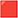	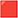	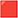						
Sudan	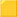 Q4 2025	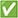	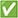	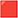	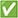	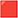	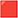	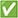			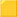 Partial		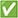
Syria	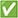	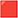	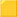 2026	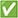	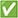	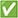	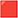	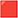					
Tunisia	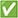	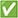	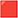	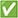	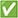	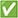	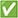	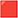	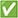				
UAE	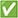	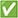	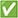	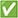	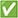	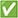	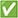	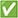	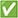	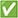			
Yemen	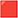	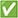	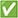	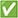	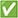	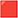	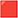	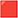					

This is a narrative review to address the question of what the impact of introduction of pneumococcal conjugate vaccines (PCVs) in children in Eastern Mediterranean Region (EMR) and the accompanied change on AMR patterns is. The primary objective of this study to investigate the serotype replacement associated with PCV introduction in the EMR and the associated trend in AMR. Based on this synthesis, the review aims to provide evidence-based insights to inform vaccine policy, including the potential need for broader-coverage vaccines, and to highlight critical gaps in surveillance and intervention.

Through identifying the extent of serotype replacement and resistance patterns, this review aims to assess the role of vaccination in mitigating AMR in *S. pneumoniae*, highlight the potential gaps in vaccine serotype coverage contributing to resistance trends and provide evidence-based recommendations for broader vaccine coverage or alternative interventions to reduce AMR in pediatric populations.

A structured search was conducted on PubMed using a PICO-informed strategy to identify relevant English-language studies. Titles and abstracts were screened for relevance, focusing on studies reporting serotype distribution, AMR data, and/or vaccine coverage related to PCVs in the pediatric populations of EMR countries. Given the narrative and exploratory nature of this review, designed to map the available evidence and discuss themes across diverse settings, allowing for the inclusion of a wide range of study designs (e.g., cross-sectional, surveillance, retrospective analyses) to comprehensively capture the regional landscape. Data on PCV introduction timelines were supplemented from WHO sources, and AMR context was drawn from the WHO GLASS reports.

This review’s findings provide valuable insights into optimizing vaccine strategies, addressing gaps in serotype coverage, and developing targeted interventions to mitigate AMR in pediatric populations in the EMR. The EMR faces unique public health challenges due to varying vaccine implementation strategies and accordingly inconsistent vaccination uptake, socioeconomic disparities, and AMR burdens. This is additionally important among children, a vulnerable population with developing immune systems and a primary target for vaccination programs.

## Main findings

### Importance of incorporating PCV into all national immunization programs

A cross-sectional study in Saudi Arabia between 2000 and 2004 showed that 54% of invasive isolates collected from children have reduced susceptibility to penicillin, with resistance rates of 26% to erythromycin and 6% to cefotaxime. On the other hand, the estimated serotype coverage of the PCV7 vaccine among children under 5 years in Saudi Arabia is 62%, increasing significantly to 83% in children under 2 years for IPD isolates. This highlights the critical role of PCV7 in reducing antibiotic-resistant infections (Shibl, 2008).

It is not only the PCV introduction, but also choosing a PCV covering the most prevalent serotypes. A cross-sectional study investigate the nasopharyngeal carriage isolates in Palestine between 2012 and 2013 discussed that among the isolated strains from Palestinian children, 37.1% were covered by the PCV7 vaccine, while 56.1% were covered by the PCV13 vaccine. The six most prevalent serotypes—19F, 6A, 23F, 6B, 19A, and 14—accounted for over half (51.6%) of the pneumococcal strains, and 80.8% of these strains were non-susceptible to penicillin (Nasereddin et al., 2013).

Another cross-sectional study in Egypt recruited 334 children (6 months–5 years old) to test for *S. pneumoniae* in Nasopharyngeal (NP) samples between 2015 and 2017 to investigate both nasopharyngeal carriage and invasive isolates. From the detected isolates, 77.4% of colonizing and 75% of invasive isolates are covered by PCV13 vaccine. Isolates detected from IPD (40 sample) showed resistance genes for penicillin in 27.5% and for erythromycin in 42.5% of the IPD isolates. This highlights the importance of addressing barriers to vaccine introduction in low- and middle-income countries and incorporating PCV into Egypt’s national immunization program (El-Kholy et al., 2020).

### PCV serotype coverage and serotype replacement

Although it is important to incorporate PCV in national immunization programs, it is additionally important to monitor the serotype pattern before and after introduction. In Lebanon, the prevalence of vaccine serotypes decreased after the introduction of PCVs. On the other hand, non-vaccine serotypes (NVTs) increased especially 24 and 16F. Additionally, AMR decreased significantly after the introduction of PCVs, for serotypes included in the vaccine. However, NVTs showed increasing resistance to erythromycin and other antibiotics. Surprisingly, mortality rates increased over the three study eras, even in vaccinated age groups (≤5 years) This unexpected increase in mortality, despite reductions in vaccine-type disease, underscores the significant disease burden that can be imposed by emerging NVTs, particularly in settings where they may exhibit enhanced virulence or AMR. It highlights that the net public health benefit of vaccination depends not only on reducing vaccine-type disease but also on closely monitoring and mitigating the impact of replacement serotypes (Reslan et al., 2022).

A multicenter, prospective observational study in Oman conducted between 2014 and 2016 post PCV13 introduction concluded that the distribution of vaccine versus non-vaccine serotypes was more pronounced in children aged ≤ 5 years, with 26.5% of isolates being vaccine serotypes and 73.5% being non-vaccine serotypes. This data highlights the prevalence of non-vaccine serotypes in the pediatric population, post vaccination, and underscores the effect of serotype replacement (Al-Jardani et al., 2019).

Later, a prospective study for children 6–36 months old visiting a pediatric center at Morocco in 2018 investigated the nasopharyngeal colonization of *S. pneumoniae* in children with acute otitis media (AOM). The study found that the pneumococcal carriage rate among children with AOM was 49.7%. Of the 63 serotyped isolates, 81% were NVTs, with the most common being 6C/6D, 10, and 19B/19C. The study also observed significant resistance to penicillin G (27.5%), while antibiotic susceptibility was higher for most other antibiotics tested, except for penicillin G and amoxicillin (Amari et al., 2022).

Another observational study in Pakistan between 2006 and 2020 examined capsular switching, and AMR profiles of *Streptococcus pneumoniae* isolates pre and post introduction of PCV10. For the AMR profiles, the study found high resistance against common antibiotics, particularly penicillin (58.9%), erythromycin (29.5%), and tetracycline (53.2%). For vaccination coverage, the study reported that the PCV10 vaccine had limited coverage of circulating serotypes in Pakistan. In disease-causing isolates, only 33.3% were covered by PCV10. Additionally, several non-vaccine serotypes, such as 6A, 3, 19A, and 23B, emerged as prevalent in the post-PCV10 era, highlighting the issue of serotype replacement and that introduction of more serotypes, as seen with PCV13 and the emerging PCV24, would improve coverage and potentially reduce disease burden (Javaid et al., 2024).

### PCV introduction and various AMR trends

The introduction of PCV and the resulted serotype replacement is accompanied by a clinical outcome and reflection on AMR trends that we should be monitoring to address promptly. A retrospective study between (2010 and 2015) in Saudi Arabia aimed to monitor trends in AMR. It reported a notable improvement in penicillin susceptibility, increasing from 37% in the pre-PCV13 vaccination period to 51% during the transitional period, and reaching 100% in the post-PCV13 vaccination period. Their finding demonstrated improved penicillin susceptibility. This outcome underscores the influence of the post-vaccination era, as vaccination has been well-documented to significantly reduce the nasopharyngeal carriage rate and IPD caused by vaccine serotypes in children, while also promoting herd immunity in adults (Farah et al., 2019).

In another surveillance study in Iran conducted between 2018 and 2019, Nearly half of the *S. pneumoniae* strains were classified as multidrug-resistant (MDR). Non-vaccine serotypes accounted for 16% of isolates, 65% of which were identified as MDR. The rising incidence of MDR serotypes, such as 19A, and increasing AMR rates is noticed (Habibi Ghahfarokhi et al., 2020). Another observational study in Iran between 2011 and 2013 focused on erythromycin resistant *S. pneumoniae* (ERSP) genes post PCV era found that 83% of the ERSP isolates were MDR (resistant to 3 or more classes). Both studies highlight the importance of continuous surveillance to monitor MDR with the introduction of various vaccines (Talebi et al., 2016).

Similarly, A retrospective analysis in UAE from 2010 to 2021 found that after introduction of PCV-13 which was introduced in the UAE in 2011, a statistically significant decline in resistance to amoxicillin and penicillin G was observed at both non-meningitis and meningitis breakpoints. On the other hand, the proportion of MDR isolates, defined as resistance to three or more antibiotic classes, increased from 16.4% in 2013 to 42.2% in 2021, with this upward trend being statistically significant. Again, this highlights how the introduction of vaccines targeting specific serotypes may lead to an increase in non-vaccine serotypes, some of which may exhibit AMR (Senok et al., 2023).

This is also supported by a cross-sectional study conducted in 2022 in Aden, Yemen. The PCV13 vaccination coverage in the study population was 76%. Among the pneumococcal isolates, 80.2% of serotypes were covered by the PCV13 vaccine in asymptomatic children, compared to 71.8% in symptomatic children. Common serotypes included 19, 1, 4, 5, and 2. Pneumococci were resistant to penicillin (96.5%), cefepime (15.8%), ceftriaxone (16.4%), and amoxicillin-clavulanate (0%). On the other hand, Erythromycin, azithromycin, and doxycycline had resistance rates of 48, 31, and 53.3%, respectively. The study found high MDR rates, with 85.7% of isolates showing resistance to at least 7 antibiotics at the meningitis breakpoint (Matran et al., 2024).

## Discussion

PCVs, particularly PCV13, have played a critical role in reducing the incidence of vaccine-type IPD. Studies in Lebanon and Saudi Arabia have reported significant reductions in vaccine-type AMR and invasive disease post-PCV introduction. However, these benefits are contingent upon vaccine coverage, which remains inconsistent across the EMR.

**APPENDIX FIGURE 1:**
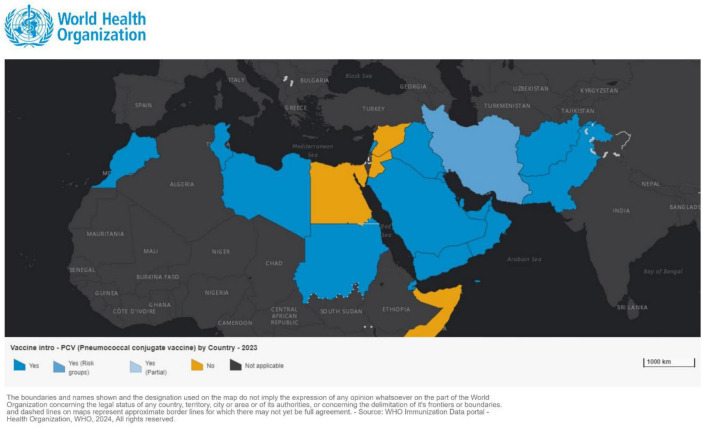
Pneumococcal conjugate vaccine introduction in Eastern Mediterranean Region in 2023.

Inconsistent vaccination coverage in many countries in EMR is exacerbated by many factors including conflict, political instability, and inadequate healthcare infrastructure. In Yemen, for example, the ongoing conflict has severely disrupted vaccination campaigns, leading to gaps in coverage, particularly in rural and conflict-affected areas. Additionally, Morocco, Tunisia, and Occupied Palestinian territory still have PCV10 instead of PCV13 in their national program. Moreover, some countries including Egypt are yet to introduce the vaccine into their national program as shown in Appendix Figure 1 (World Health Organization [WHO], 2024b).

To contextualize pneumococcal conjugate vaccine adoption across the EMR, Table 2 presents the timeline of national PCV introductions up to 2025 based on WHO EMRO datasets.

**TABLE 2:** PCV type and year of introduction in Eastern Mediterranean Region.

Country	Year of PCV introduction	Current PCV type in use
Qatar	2005	PCV13
United Arab Emirates	2007	PCV13
Kuwait	2007	PCV13
Oman	2008	PCV13
Bahrain	2008	PCV13
Saudi Arabia	2009	PCV13
Palestine (Occupied Territories)	2009	PCV13
Morocco	2010	PCV13
Yemen	2011	PCV13
Pakistan	2012	PCV10
Afghanistan	2013	PCV13
Libya	2013	PCV13
Sudan	2013	PCV13
Lebanon	2015	PCV13
Iraq	2017	PCV13
Tunisia	2019	PCV13
Djibouti	2021	PCV13
Egypt	Not yet	—
Iran	Planned 2024 (phased)	PCV10
Jordan	Planned 2025	PCV13
Somalia	Not yet	—
Syria	Not yet	—

Serotype replacement is a well-documented challenge following PCV introduction. NVTs, such as 24 and 16F in Lebanon, and 6C and 19B in Morocco, have emerged as significant contributors to disease, AMR, and higher mortality. The increase in disease and mortality, despite reductions in vaccine-type disease, underscores the significant disease burden that can be imposed by emerging NVTs, particularly in settings where they may exhibit enhanced virulence or AMR. It highlights that the net public health benefit of vaccination depends not only on reducing vaccine-type disease but also on closely monitoring and mitigating the impact of replacement serotypes. Ongoing surveillance and adaptation of vaccine strategies are crucial to mitigate the public health impact of NVTs. Additionally, there is a need for broader serotype protection and targeted interventions. The introduction of higher-valency vaccines, such as PCV15 or PCV20, could further address gaps left by serotype-specific limitations of PCV13.

The review reveals a recurring pattern: a decline in resistance among vaccine-targeted serotypes coupled with the emergence and spread of NVTs that frequently exhibit high-level and MDR. This pattern can be mechanistically explained by a combination of serotype replacement and clonal expansion. PCV application selectively removes vaccine-type strains, creating an ecological niche filled by NVTs. Some of these NVTs belong to genetic clones with a high propensity for AMR and capsule-switching (e.g., the globally disseminated MDR related to serotype 19A). Furthermore, persistent high antibiotic consumption in the region continues to exert selective pressure, favoring the survival and spread of these resistant NVT clones.

**APPENDIX FIGURE 2:**
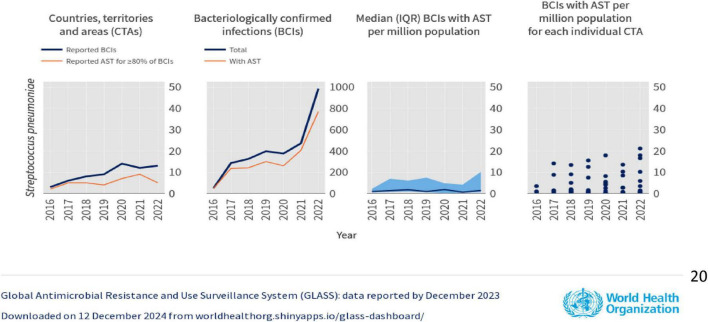
Testing coverage in blood stream infection by S. Pneumonia in Eastern Mediterranean Region 2016–2022.

**APPENDIX FIGURE 3:**
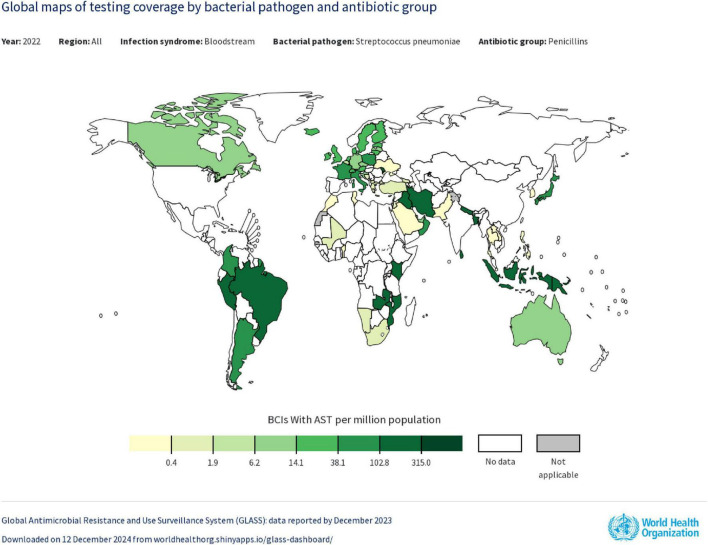
Global maps of testing coverage by Streptococcus Pneumonia and Penicillin in 2022.

Additionally, despite progress in reducing vaccine-type AMR, overall trends in AMR remain concerning across the region. In country such as Pakistan, resistance to penicillin, macrolides, and other antibiotics persists, even post-PCV introduction. Capsular switching and the persistence of MDR strains complicate efforts to combat AMR. Surveillance studies in Saudi Arabia and Morocco demonstrate that while reductions in penicillin resistance are possible, serotype-specific challenges remain. Appendix Figures 2, 3 are showing the increase in resistance pattern over the past years and the intensity of s. pneumonia resistant to penicillin in the region (World Health Organization [WHO], 2024a). Strengthening antibiotic stewardship programs and integrating these efforts with vaccination strategies are essential to addressing the AMR burden. This is concerning given that pneumonia, a leading cause of morbidity and mortality, is often caused by *S. pneumoniae* strains resistant to treatment.

Despite documented progress in reducing vaccine-type AMR, overall resistance trends in the EMR remain complex and concerning. The comparability of MDR trends across studies is limited by heterogeneous definitions, but the consistent signal of rising MDR is clear. This is particularly alarming as pneumonia, a leading cause of death, is often caused by S. pneumoniae strains that may be increasingly difficult to treat.

The impact of PCVs on AMR is highly heterogeneous across the EMR, largely determined by underlying coverage and context. Countries with sustained, high PCV coverage and stronger health systems demonstrate the most pronounced reductions in vaccine-type disease and associated AMR. In contrast, countries with low, inconsistent coverage or those yet to introduce PCV (e.g., Egypt, conflict-affected states) continue to bear a high burden of vaccine-preventable disease and AMR. Conflict and political instability, as in Yemen, Syria, and Libya, severely disrupt vaccination programs and surveillance, creating large immunity gaps and data voids that obscure the true burden of disease and resistance. This lack of data from the most vulnerable settings is a critical limitation of the regional evidence base and likely leads to an underestimation of both disease and AMR challenges. While the discussed country-specific studies are heterogeneous in design and setting, reveal several overarching patterns regarding serotype coverage, replacement, and AMR trends post-PCV introduction in the EMR, as per examples summarized in Table 3.

**TABLE 3:** Summary of select country studies on serotype coverage and AMR trends post-PCV introduction in the EMR.

Country (PCV type)	Key finding on serotype coverage/replacement or AMR trend post-PCV
Saudi Arabia (PCV13)	• PCV7 coverage: 62% (<5y), 83% (<2y) for IPD. • Penicillin susceptibility in IPD isolates improved from 37% (pre-PCV13) to 100% (post).
Palestine (PCV13)	• PCV7 coverage: 37.1%; PCV13 coverage: 56.1% of carriage isolates. • 80.8% of the six most prevalent serotypes were penicillin-non-susceptible.
Egypt (Pre-PCV)	• PCV13 covers 77.4% of colonizing and 75% of invasive isolates. • Penicillin and erythromycin resistance genes found in 27.5 and 42.5% of IPD isolates.
Lebanon (PCV13)	• Vaccine serotypes decreased; NVTs (24, 16F) increased with PCV introduction. • AMR decreased for vaccine types but increased for NVTs. Mortality increased.
Oman (PCV13)	Post-vaccine: 26.5% vaccine vs. 73.5% non-vaccine serotypes in ≤ 5y IPD.
Pakistan (PCV10)	• PCV10 covers only 33.3% of disease-causing isolates. NVTs (6A, 3, 19A) emerged. • High pre-existing resistance to penicillin (58.9%), erythromycin (29.5%).
UAE (PCV13)	Significant decline in penicillin/amoxicillin resistance. MDR proportion increased from 16.4% (2013) to 42.2% (2021).
Yemen (PCV13)	• PCV13 covers 80.2% (asymptomatic) vs. 71.8% (symptomatic) carriage isolates. • Extremely high penicillin resistance (96.5%); 85.7% MDR (≥7 antibiotics).

This narrative was limited by inconsistencies in regional surveillance data quality and methodological heterogeneity across included studies (e.g., varying MDR definitions: ≥ 3 vs. ≥ 7 antibiotic classes; cross-sectional vs. prospective designs). Underreporting, especially from conflict-affected countries, and reliance on older diagnostic techniques may have influenced findings. Publication bias is possible, as studies reporting positive PCV outcomes (e.g., serotype coverage/AMR reductions in high-income EMR countries like Saudi Arabia/UAE) are more likely published than null/negative results from resource-constrained settings. Critically, absent or sparse data from conflict-affected countries (Syria, Somalia, Sudan) underestimates true regional AMR burden and serotype replacement dynamics, as ongoing instability disrupts surveillance and vaccination, potentially masking higher NVT-MDR prevalence.

Additionally, heterogeneity in vaccine introduction timelines complicates direct comparisons between countries. Another significant challenge in many EMR countries is the lack of robust surveillance systems to monitor both AMR trends and PCV coverage, which is either insufficient or outdated, hindering efforts to assess the true burden of disease and track the effectiveness of vaccination programs.

## Conclusion

This review highlights the pivotal role of pneumococcal conjugate vaccines in reducing vaccine-type pneumococcal diseases and associated AMR. However, serotype replacement and suboptimal vaccine coverage remain significant challenges, particularly in conflict-affected and resource-limited settings. Broader serotype coverage and enhanced surveillance are essential to address these gaps.

### Future recommendations

To maximize PCV impact on pediatric AMR in resource-constrained EMR settings, we propose the following prioritized, actionable strategies:

- *Strengthen sentinel AMR & serotype surveillance systems (highest priority)*: Robust surveillance systems collecting timely and accurate data on pneumococcal infections, AMR patterns, and vaccine coverage.
- *Increase PCV coverage*: Improve coverage and use of higher-valency conjugate vaccines. Strategies should include mobile vaccination units, outreach programs, and public awareness campaigns to increase parental confidence in vaccination. Special efforts should be done to ensure that children in high-risk populations, including those living in overcrowded, poor conditions, and conflict affected areas are vaccinated.
- *Monitor serotype replacement*: Ongoing monitoring of pneumococcal serotype distribution is crucial to detect any shifts toward non-vaccine serotypes. Data on serotype replacement should be integrated into vaccination policy discussions to ensure that PCV remains effective in preventing pneumococcal infections and reducing AMR.
- *Enhance antibiotic stewardship*: Healthcare providers should be trained on appropriate antibiotic prescribing practices, and public health campaigns should emphasize the importance of using antibiotics only when necessary.
- *Promote regional and international collaboration*: Collaboration between countries in the EMR and with international organizations can help share knowledge, resources, and expertise in tackling AMR and improving vaccination coverage.
- *Address socio-economic determinants****:*** improving access to healthcare, addressing malnutrition, and reducing overcrowding, to reduce the spread of pneumococcal disease.

## Appendix

### PICO

**Population:** Children in EMR

**Intervention/exposure:** Conjugate pneumococcal vaccine CPV7, 10, and 13 (As the provided in EMR)

**Comparison:** pre and post introduction of different CPVs

**Outcome:** Trends in antimicrobial resistance

**Design:** No limit. I’m interested in exploring the effect of vaccination itself regardless of the study design whether observational or interventional study.

### Research strategy

The following research is done on PubMed:

(“pneumococcal” OR “*Streptococcus pneumoniae*” OR “*S. pneumoniae*” OR “otitis media” OR “acute otitis media” OR “OM” OR “AOM” OR “invasive pneumococcal disease” OR “pneumococcal disease” OR “pneumonia” OR “pneumococcal pneumonia”) AND (“Drug Resistance, Bacterial” [MeSH] OR “drug resistance: OR “Antibiotic resistance” OR “Antimicrobial resistance” OR “Resistance” OR “Antibiotic” OR “Antimicrobial” OR “AMR”) AND (“Vaccination”[MeSH] OR “Immunization Programs”[MeSH] OR “Vaccination” OR “Vaccine” OR “PCV” OR “PCV7” OR “PCV10” OR “PCV13” OR “Pneumococcal Conjugate Vaccine” OR “Coverage” OR “Uptake” OR “Vaccination rate”) AND (“Child”[MeSH] OR “Infant”[MeSH] OR “children” OR “pediatrics” OR “paediatrics” OR “infants”) AND (English[All Fields])) AND ((“Eastern Mediterranean Region”[Title/Abstract] OR “Middle East”[Title/Abstract] OR Afghanistan[Title/Abstract] OR Bahrain[Title/Abstract] OR Djibouti[Title/Abstract] OR Egypt[Title/Abstract] OR Iran[Title/Abstract] OR Iraq[Title/Abstract] OR Jordan[Title/Abstract] OR Kuwait[Title/Abstract] OR Lebanon[Title/Abstract] OR Libya[Title/Abstract] OR Morocco[Title/Abstract] OR Oman[Title/Abstract] OR Pakistan[Title/Abstract] OR Palestine[Title/Abstract] OR west bank[Title/Abstract] OR Qatar[Title/Abstract] OR Saudi Arabia[Title/Abstract] OR Somalia[Title/Abstract] OR Sudan[Title/Abstract] OR Syria[Title/Abstract] OR Tunisia[Title/Abstract] OR United Arab Emirates[Title/Abstract] OR Yemen[Title/Abstract]))

Title and abstract screening are done to choose the relevant articles and 20 study is included based on the study relevant to the chosen themes.

Additionally, vaccination data regarding PCV introduction in EMR from WHO website and AMR data from GLASS website (Global Antimicrobial Resistance and Use Surveillance System).
